# Preventive Effect of Glycyrrhiza Glabra Extract on Oral Mucositis in Patients Under Head and Neck Radiotherapy: A Randomized Clinical Trial

**Published:** 2017-09

**Authors:** Shamsolmolok Najafi, Soraiya Ebrahimpour Koujan, Soheila Manifar, Mohammad Javad Kharazifard, Saba Kidi, Samira Hajheidary

**Affiliations:** 1 Assistant Professor, Department of Oral Medicine, School of Dentistry, Tehran University of Medical Sciences, Tehran, Iran; 2 PhD Student, Department of Community Nutrition, School of Nutritional Sciences and Dietetics, Tehran University of Medical Sciences, Tehran, Iran; 3 Epidemiologist, Dental Research Center, Dentistry Research Institute, Tehran University of Medical Sciences, Tehran, Iran; 4 Dentistry Student, School of Dentistry, International Campus, Tehran University of Medical Sciences, Tehran, Iran; 5 Postgraduate Student, Department of Periodontics, School of Dentistry, Tehran University of Medical Sciences, Tehran, Iran

**Keywords:** Mucositis, Glycyrrhiza, Radiotherapy, Mouth Neoplasms

## Abstract

**Objectives:**

About two-thirds of cancer patients undergo radiotherapy. Oral mucositis represents a major complication of radiotherapy, causing morbidity and mortality and decreasing the quality of life of patients. This study aimed to assess the preventive effect of Glycyrrhiza aqueous extract on oral mucositis in cancer patients under head and neck radiotherapy.

**Materials and Methods:**

In this double-blind clinical trial, 37 head and neck cancer patients were divided into intervention (n=19) group receiving Glycyrrhiza aqueous extract and control (n=18) group receiving placebo. Patients in the test group used Glycyrrhiza aqueous extract topically twice a day from the first day of starting radiotherapy until the end of the second week. Patients were examined in the first day of radiotherapy for any type of wound before treatment and those with oral ulcers before radiotherapy were excluded from the study. The grade of mucositis was determined using the classification by the World Health Organization. ANCOVA was performed to assess any difference between the two groups with regard to oral mucosal irritation and wound size after the intervention while controlling for the covariates such as sex and age.

**Results:**

Significant differences were found in the maximum grade of mucositis and oral mucosal irritation between the intervention and control groups (P<0.001).

**Conclusions:**

This study showed that aqueous extract of Glycyrrhiza can be effective for decreasing the severity of oral mucositis in head and neck cancer patients undergoing radiotherapy.

## INTRODUCTION

Head and neck cancer is common worldwide. Cancers of the oral cavity account for about 10% of all malignancies in males and 4% in females [1–3]. The commonly used treatment modalities for head and neck cancer include surgery, radiotherapy and chemotherapy. Radiotherapy has complications such as mucosal inflammation, periodontal infection, fungal and viral infections and salivary gland dysfunction [4]. Mucosal inflammation or oral mucositis is the main complication of radiotherapy of the head and neck region, which is painful and starts in early phases of treatment. It becomes more severe by continuation of treatment. This condition may become so severe to discourage patients from continuing treatment [5, 6]. Factors affecting the duration and degree of mucositis in these patients include the radiation source, cumulative dose, its intensity, volume of irradiated mucosa, smoking and alcohol consumption as well as predisposing factors such as xerostomia or infection [7, 8]. In addition, no definite method has been established for prevention and treatment of oral mucositis in these patients, but prophylactic or therapeutic elimination of pathogenic flora is often performed by use of antiseptic and antimicrobial agents and different mouthwashes to alleviate the pain and resolve oral mucositis [8, 9]. However, these modalities are not effective and may even cause side effects [9]. Considering the significance of this matter, modalities are needed for prevention and control of oral mucositis [9]. Recently, herbal medicine has been recommended for prevention and treatment of diseases worldwide due to lower complications [10]. Licorice (Glycyrrhiza glabra L., from the Papilionaceae/Fabaceae family) is a traditional medicinal herb, which grows in various parts of the world. It is a very sweet, moist and soothing herb. The roots are long, cylindrical, thick and multi-branched [10]. It has been reported that it has antimicrobial, anti-inflammatory [10] and antiviral [11] properties. A root component (glycyrrhizin) is being generally regarded as the major biologically-active component. Glycyrrhiza glabra root includes the following components: 20% moisture, 5–24% glycyrrhizin (a triterpene glycoside considered to be the main biologically-active component), 3–16% sugar, 30% starch and 6% ash. However, the exact composition varies greatly according to species, cultivation conditions and growth environment [11]. Licorice is used to relieve inflammation, throat infection, gastric and peptic ulcers, arthritis, eye and liver diseases and sex-hormone imbalance. Also, it has anti-Helicobacter pylori and antibacterial activities [12]. Given that the positive efficacy of Glycyrrhiza aqueous extract for prevention of oral mucositis is confirmed, it may be used for this purpose to increase patient tolerance in order to continue radiotherapy.

To the best of authors’ knowledge, no previous study in Iran has been conducted on this topic. Therefore, this study was conducted to assess the efficacy of Glycyrrhiza aqueous root extract for prevention of oral mucositis in patients under head and neck radiotherapy.

## MATERIALS AND METHODS

### Study population:

**Fig. 1:**
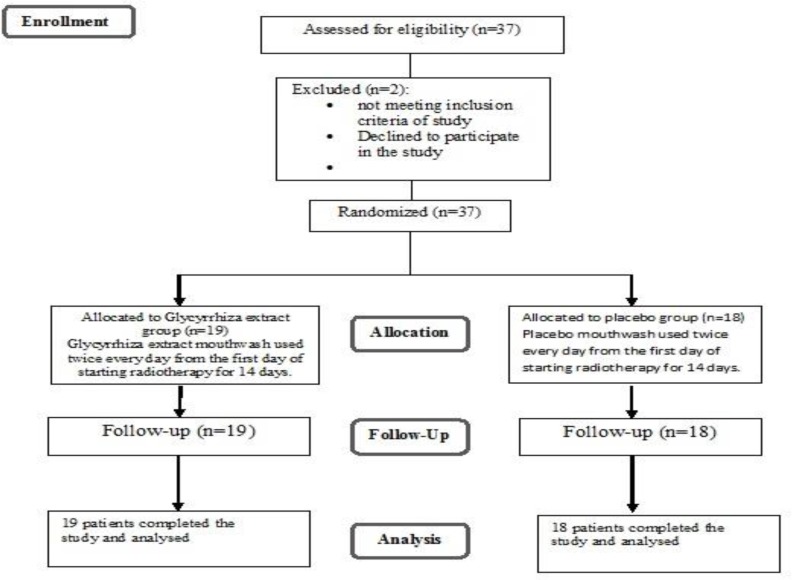
Flowchart of the study design

We recruited 37 patients under radiotherapy in the Oncology Ward of Khoramabad Ashayer Shohada Hospital and cancer institute of Imam Khomeini Hospital who participated in this study from October 2010 to October 2011. The inclusion criteria were as follows: Patients had to be at least 18 years old with definite diagnosis of head and neck cancer. The radiation dose determined by the radiotherapist had to be at least 50 Gy (the average dose that often causes mucositis), patients had to be able to use the extract topically before each radiotherapy session and at least 50% of the oral cavity had to be under irradiation (according to the radiotherapist). The exclusion criteria were defined as patients who were already under radiotherapy for the head and neck cancer and/or had received any type of treatment or radiation therapy for the head and neck cancer in the past year, having allergy to Glycyrrhiza, or showing side effects to it. Patients who could not use aqueous Glycyrrhiza extract according to their attending physician, had an active oral infection or oral mucosal wound before starting radiotherapy, those unwilling to participate or remain in the study at any step of treatment and patients with poor oral hygiene were also excluded (Fig. 1).

Sample size was determined to be 18 samples in each group considering the frequency of mucositis following radiotherapy without intervention to be 95% and assuming a decline in the rate of severe mucositis by up to 55% as well as 40% difference between the two groups by confidence interval of 95%, α=0.05 and power of 80%.

### Study design:

The present study was a randomized placebo-controlled double-blind clinical trial, which was conducted in accordance with the guidelines of the Declaration of Helsinki (1964), and was approved by the ethics committee of Tehran University of Medical Sciences (no. 92.130.1551). The study was registered in the Iranian Registry of Clinical Trials (available at: http://www.irct.ir, identifier: IRCT201203012464N4). All participants provided written informed consent prior to treatment and were given a copy of the signed informed consent document. The examiner evaluated them at the end of the second week of radiotherapy. All patients and the examiner were blinded to the intervention. Thirty-seven eligible patients were randomly divided into two groups using block randomization (Random Allocation Software: RAS) [12]. There were 19 patients in the intervention and 18 patients in the control group. Our primary outcomes were the incidence of radiation-induced oral mucositis, the secondary irritation and oral mucosal wound size. The experimental group received 100 cc of 50% aqueous extract of Glycyrrhiza (extracted with alcohol and water). The placebo group was given the same amount of placebo containing approved brown food dye in 2 L of water.

The aqueous Glycyrrhiza extract and the placebo were the same in terms of appearance, color and taste. The bottles containing 100 cc of 50% aqueous extract of Glycyrrhiza or placebo were given to patients and they were asked to use 20 cc twice per day for 14 days after starting radiotherapy. All patients were also instructed to clean their teeth with a soft toothbrush three times a day (after every meal). They received pretreatment dietary counseling and dental evaluation which was reinforced weekly. The prescription dose was determined by the physician and based on the objectives of treatment, tumor stage, and the patient’s general condition. The total radiotherapy doses ranged from 50–60 Gy in this study.

### Clinical evaluation:

All patients were examined in the first day of radiotherapy and were checked for every type of wound before radiotherapy. Patients who had oral ulcers before radiotherapy were excluded from the study. At the 14th day of radiotherapy, the oral cavity was checked for any sign of mucositis.

### Clinical diagnosis of oral changes was made based on the following criteria:

Mucositis: mucous membrane erythema that is sometimes associated with irritation, erosion or wound. Moreover, mucositis was checked based on the World Health Organization criteria and clinical evaluation (Table 1). After evaluation of the oral cavity, the grade of radiation-induced oral mucositis was scored by one examiner. The patients’ perspective of irritation within the radiotherapy field was assessed using a 10 cm visual analog scale (VAS) at the 14th day of radiotherapy. Oral mucosal wound size was defined as measuring the largest diameter of oral ulcer (in millimeters) evaluated by clear flexible scaled ruler.

**Table 1.** mucositis grading according to the World Health Organization

**Grade**	**Definition**
0	Mucous membrane without change
1	Mucous membrane has mild inflammation and sometimes mild pain that does not need anti-inflammation medication
2	Point mucositis associated with mild serous, inflammatory discharge and mild pain that needs pain killer
3	Diffused and continuous mucositis with fibrous discharges and severe pain that needs pain killer
4	Wound and bleeding or necrosis

### Extract preparation and authentication:

Glycyrrhiza glabra roots were collected from Kordestan, Iran. The pharmacognostical study of the drug was performed in the pharmacognosy laboratory, Institute for Post Graduate Teaching and Research in the Faculty of Pharmacology at Tehran University of Medical Sciences.

### Method of preparation of Glycyrrhiza glabra root extract

The freshly prepared 500 g of Glycyrrhiza glabra root powder was poured into a 25. L beaker, and 2 L of distilled water was added to it. The mixture was stored overnight at room temperature and then the extract was isolated and filtered using a thin cotton cloth. This was repeated three times. The extract was placed in a rotary vacuum distiller to obtained a resin like mixture. The mixture was refrigerated away from direct light.

### Statistical analysis:

All data analyses were performed by SPSS software version 20.0 (SPSS Inc., Chicago, IL, USA). The results were expressed as mean ± standard deviation (SD). The normal distribution of data was assessed by one-sample Kolmogorov-Smirnov test. Demographic characteristics of the patients in the two groups were compared using the chi-square test or independent t-test for qualitative and quantitative variables, respectively. ANCOVA was performed to assess any difference between the two groups with regard to mucosal irritation and wound size after the intervention, controlling for covariates such as sex and age. The Mann-Whitney test was used to identify any difference between the groups in terms of mucositis.

## RESULTS

General characteristics of the study population are shown in Table 2. There were no statistical differences between the intervention and control groups in terms of demographics and clinical status at baseline. In brief, 37 patients with one type of head and neck cancer participated in this study. Among those, 10 patients (27%) had squamous cell carcinoma (SCC) of the larynx, six patients (16%) had SCC of the tongue, twopatients (5%) had lip cancer, six patients (16%) had SCC of the hypopharynx, three patients (8%) had neck cancer, four patients (10%) had buccal SCC, one patient (5%) had SCC of the ear, one patient (5%) had SCC of the nasopharynx and four patients (10%) had SCC of the nose. Table 3 shows the effects of aqueous Glycyrrhiza extract on mucositis after 14 days of use. Comparison of the maximum grade of mucositis in the two groups showed that among the intervention group patients, 12 (63.15%) suffered from mucositis grade I and seven patients (36.84%) had mucositis grade II. Mucositis grade III was not seen in any patient in this group. In the placebo group, one patient (5.55%) had mucositis grade I, six patients (33.3%) had mucositis grade II and 11 patients (61.11%) had mucositis grade III. Mucositis grade IV was not seen in any patient. The difference in the grade of oral mucositis was significant between the two groups (P<0.001).

**Table 2.** General characteristics of patients under head and neck radiotherapy at baseline

**Variable**	**Glycyrrhiza glabra group (n=19)**	**Placebo group (n=18)**
**Age (yrs.)**	47.05±10.75	49.72±9.72
**Male/Female N (%)**	14 (74)/5 (26)	10 (56)/8 (44)
**Radiotherapy dose (Gy)**	53.68±4.95	53.88±5.01

Values are presented as mean ± standard deviation except sex that presented as number (percentage). For all characteristics, there were no significant differences between the Glycyrrhiza glabra and placebo groups (all Ps>0.05, based on independent samples t-test for quantitative variables and chi-square test for sex).

**Table 3.** Degree of mucositis in patients under head and neck radiotherapy after 14 days of Glycyrrhiza glabra use

**Mucositis degree (N/%)**	**Glycyrrhiza glabra group (n=19)**	**Placebo group (n=18)**	P value^*^
Grade I	12 (63.2)	1(5.6)	<0.001
Grade II	7(36.8)	6(33.3)	
Grade III	0	11(61.1)	

Data were presented as number (percentage).

* P value is reported based on the analysis of Mann-Whitney U test.

**Fig. 2:**
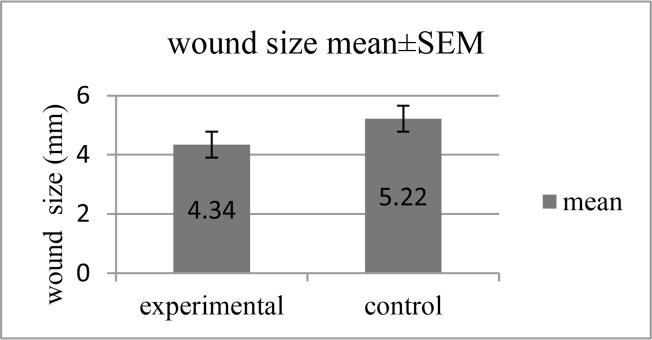
Effect of aqueous Glycyrrhiza extract on wound size

**Fig. 3:**
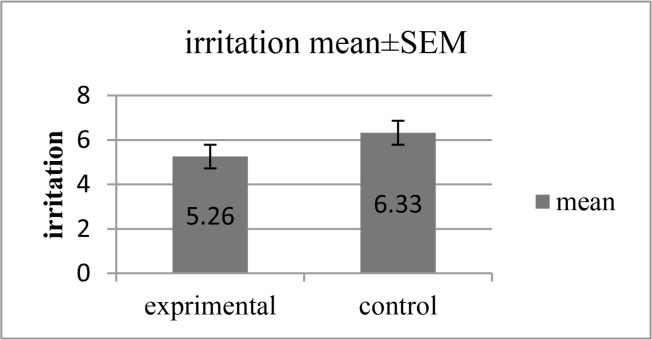
Effect of aqueous Glycyrrhiza extract on oral mucosal irritation

Table 4 shows the effect of aqueous Glycyrrhiza extract on mucosal irritation and mucosal wound size. The mean score of oral mucosal irritation and oral mucosal wound size was 5.26 and 6.33 in the intervention group and 4.34 and 5.22 in the placebo group, respectively. The results of independent sample-t test showed that there were statistically significant differences between the two groups in terms of the two variables mentioned above (P=0.03 and P= 0.04, respectively). These results were significant after adjusting for major confounders such as sex and age by ANCOVA (P =0.05 and P=0.03, respectively; Figs. 2 and 3).

**Table 4.** Irritation and wound size in patients under head and neck radiotherapy after 14 days of using Glycyrrhiza glabra

**Variable**	**Glycyrrhiza glabra group (n=19)**	**Placebo group (n=18)**	**MD(95%CI)**	**P value**
**Irritation**	5.26±1.55	6.33±1.32	−1.07 (−2.03, −0.10)	0.03^a^
			−1.02 (−2.04, −0.002)	0.050^b^
**Wound size (mm)**	4.34±1.23	5.22±1.37	−0.88(−1.75, −0.008)	0.04^a^
			−0.97 (−1.89, −0.06)	0.03^b^

The results are described as mean± standard deviation (SD).

a Mean difference (95%CI), P value is reported based on the analysis of independent sample t-test.

b Mean difference (95%CI), P value is reported based on the analysis of covariance

## DISCUSSION

Based on our literature review, this study is the first trial on the effects of aqueous Glycyrrhiza extract mouthwash on oral mucositis, irritation and wound size in patients under head and neck radiotherapy. This study showed that topical use of aqueous Glycyrrhiza extract by patients under head and neck radiotherapy reduced oral mucositis. Also, there were differences between the intervention and control groups regarding oral mucosal irritation and oral mucosal wound size. Mucositis is a common side effect of cancer treatment resulting from the systemic cytotoxic effects of chemotherapy and the local effects of radiation directly on the head and neck region [13].

Nowadays, natural products with anti-inflammatory and/or wound healing properties could serve as agents for prevention or minimization of radiation-induced oral mucositis, and a previous study showed that several natural products possess beneficial effects suitable for amelioration of radiation-induced oral mucositis [14].

Even though the process of radiation-induced oral mucositis is not yet fully understood, itappears that these treatments, aimed at rapidly destroying the proliferating cancer cells, also destroy normal rapidly dividing cells. Tissues with high levels of mitotic activity especially mucosal membranes are significantly affected by radiation, since the most sensitive phases of the cell cycle are G2 and mitosis. They can mediate a complex of inflammatory and vascular processes in the mucosal tissue that contribute to ulceration, pain and related sequelae [15]. Glycyrrhiza glabra (Licorice) extract and its major component, glycyrrhizin, have extensive use in both traditional and herbal medicine [16]; also, glycyrrhetinic acid component that has only a few rare side effects, is generally recognized as safe by the US Food and Drug Administration [17].

The mechanism of action of Glycyrrhiza extract on mucositis may be mediated by its anti-inflammatory effects through inhibition of activated macrophages leading to inhibition of the prostaglandin E2 production, and formation of superoxide and hydroperoxide in macrophages [18]. On the other hand, anti-inflammatory effects of glycyrrhizin are probably mediated by its ability to decrease generation of reactive oxygen species by neutrophils, and direct scavenging of free radicals [19, 20]. Also, several studies have reported anti-bacterial, anti-fungal, anti-viral, anti-inflammatory and anti-allergy properties of Glycyrrhiza [21–27].

Moreover, it is used as a mouthwash for canker sore treatment, reducing its pain and healing of wounds [2]. Das et al. [28] reported the use of a mouthwash containing Glycyrrhiza glabra extract for two weeks. The treatment resulted in pain relief and accelerated the healing of aphthous ulcers. In another clinical study, patients with recurrent aphthous ulcers were assigned to receive either a patch extract of Glycyrrhiza root, a placebo patch, or no treatment at the onset of lesion. Ulcer size and pain improved with the extract when compared to the placebo and no-treatment groups [29]. Das et al, [30] in another study evaluated patients with oral mucositis induced by radiotherapy, who received local application of various substances in the oral cavity prior to their treatment. In a seven-week intervention course, group A patients applied licorice powder and honey topically on the lesions daily and used 10 mL of a licorice preparation twice a day; group B applied licorice powder and honey topically; group C applied only honey topically and group D (control) received standard medical treatment for mucositis. Group A experienced the greatest reduction in radiation- and chemotherapy-induced mucositis compared to the control group. They concluded that the intensity of radiation- and chemotherapy-induced mucositis decreased to a great extent by Glycyrrhiza glabra. These results may also indicate that the beneficial effects may also be attributed to honey. This study did not mention blinding for evaluation of mucositis [30]. This was the only study on the protective effect of Glycyrrhiza glabra on radiation-induced mucositis in the head and neck cancer patients.

There were some limitations in our study. We could not perform a parallel, randomized, controlled clinical trial and could not measure the exact baseline characteristics to explore the definite protective effects of Glycyrrhiza extract. The duration of intervention and sample size of the study were among other limitations. While studies on Glycyrrhiza (licorice) and its use during radiotherapy, in particular, are rare, it is recommended to perform similar studies on higher number of patients with a longer follow up and different dosages to better evaluate the efficacy of this extract and compare its protective effects with other treatment modalities.

## CONCLUSION

According to the results of the present study, Glycyrrhiza aqueous extract decreased the oral mucositis, wound size and irritation. These results suggest that aqueous extract of Glycyrrhiza root may be effective for reduction of oral mucositis and its complications. However, more well-designed randomized trials and large studies are warranted in this respect.
